# Inductive process of moral distress development in viewpoints from surgical nurses: a mixed-method study

**DOI:** 10.1186/s12912-024-01786-3

**Published:** 2024-03-21

**Authors:** Azam Hosseinpour, Fatemeh Keshmiri

**Affiliations:** 1 Department of Operating Room, School of Allied Medical Sciences, Qom University of Medical Sciences, Qom, Iran; 2 Medical Education Department, Education Development Center, Shahid Sadoughi University of Medical Sciences, Yazd, Iran; 3 The National Agency for Strategic Research in Medical Education, Tehran, Iran

**Keywords:** Moral distress, Nursing, Surgery, Operating room

## Abstract

**Background:**

Moral distress is a multifactorial and complex phenomenon influenced by various individual, cultural, and systemic factors. This study aimed to investigate the frequency and intensity of nurses’ moral distress, explore their experiences, and develop the conceptual model of risk factors of moral distress in surgical units and operating rooms.

**Method:**

This is a sequential mixed-method study conducted at four teaching hospitals affiliated with the Qom University of Medical Sciences. In the first step, the moral distress of nurses in surgical units and operating rooms was investigated by a survey. The participants included nurses who worked in the operating room and surgical units. (*n* = 180). The data was collected by a Moral Distress Scale-Revised (MDS-R) questionnaire. In the second step, the experiences of nurses regarding risk factors of moral distress were explored using semi-structured interviews and analyzed using the conventional content analysis by Graneheim and Lundman’s approach.

**Results:**

One hundred eighty nurses participated in this study. The mean total moral distress scores ranged from 12 to 221, with a mean (SD) of 116.8 (42.73). The causes of moral distress cited with the highest frequency and intensity related to the ‘role of healthcare providers’. The experiences of the participants in the theme ‘Inductive process of moral distress development’ were categorized into three categories: ‘Melting into the faulty system’, ‘Power and the system as distress promotors’, and ‘Perceived unpleasant consequences’.

**Conclusion:**

The results indicated that the frequency of moral distress in operating rooms and surgical units was at a moderate level and the distress intensity of nurses was at a moderately high level. The results indicated that in the investigated system, the “inductive moral process of distress development” was continuously understood by the participants. This process was influenced by systemic and individual factors. Weak assertiveness, conservative compromise, and desensitization to unprofessionalism as individual factors were effective in causing distress. Risk factors at the systemic level led nurses to melt into the faulty system and created adverse outcomes at the individual level. The lack of systemic support and the stabilization of mobbing by powerful system members had a negative impact on the individual factors of distress development. Also, these factors directly cause negative consequences.

**Supplementary Information:**

The online version contains supplementary material available at 10.1186/s12912-024-01786-3.

## Introduction

Moral distress is recognized as one of the challenges for human resources in healthcare systems [1]. Corley (1995) defined moral distress as painful feelings and/ or psychological disequilibrium caused by a situation in which one believes and knows the ethically ideal action to take but cannot carry out that action. Because they pretended to institutionalize obstacles such as lack of time, lack of supervisory support, medical power, institutional policies, or legal limitations [2]. Moral distress occurs when a provider believes that doing something ethically wrong and has little power to change the situation. This pressure to act unethically is the defining concept of moral distress [3]. Key components of moral distress are described as (a) complicity in wrongdoing, (b) lack of voice, (c) wrongdoing allied with professional values, (d) repeated experiences, and g) three levels of root causes (patient, unit, and system) [3]. The results of various studies revealed that the frequent exposure of employees to moral distress has caused adverse consequences on the performance, and psychological health of the workers, and also the healthcare outcomes of the patients [4]. Austin indicated the moral distress of physicians and nurses significantly impacted their turnover intent and professional quality of life [5].

### Moral distress in operating rooms and surgical units

Moral distress among surgeons is a complex phenomenon that is influenced by factors at multiple levels [6–8]. Fagerdahl and colleagues indicated workers in the operating room including surgeons, anesthesiologists, specialist nurses, and nurse assistants experienced moral distress because they worked hard during the COVID-19 pandemic, although their efforts were not enough according to their moral ideals. They experienced the negative stress of being in the unknown, performing work tasks in an unfamiliar place and situation, and experiencing conflict feelings of relief and guilt [9]. Exploration of the risk factors that cause moral distress, and the consequences that arise from moral distress such as burnout have an important role in planning to reduce and manage them [7, 10].

Morley et al., (2019) implied that there is little agreement about conditions that cause moral distress. Corley (2002) defined moral distress sources into two classes. The first is the internal context as nurses’ psychological response, and the second is the external context as the work environment [11]. Alimoradi and colleagues in a systematic review and meta-analysis study indicated nurses working in developing countries reported higher levels of moral distress than those working in developed countries [12]. Since moral distress was recognized as a multifactorial phenomenon, further study was suggested to explore the source of moral distress in different cultures and communities from the perspective of different professions [13, 14]. The method enables nurses to examine the processes, experiences, and outcomes of moral distress from their perspective [15]. Burton and colleagues aimed to develop an understanding of factors affecting moral distress among nurses during the COVID-19 pandemic. They used the mixed-method design due to the complexity of moral distress, and the need to rapidly gather various data, and as in-depth as possible to support the health of the nursing workforce in pandemic conditions [16]. Prompahakul and colleagues in a mixed-method study showed the seven highest-ranked causes of moral distress to categorize four system-level workload issues and three patient-level problems. They acknowledged the explored results as influenced by the culture and context of Thailand nurses [17].

The present study aimed to investigate the frequency and intensity of nurses’ moral distress in surgical units and operating rooms explore the nurses’ experiences and develop a conceptual model of risk factors of moral distress in surgical units and operating rooms.

## Method

An exploratory mixed-method sequential explanatory design was used. First, a quantitative survey was conducted to evaluate the moral distress levels of nurses. This was followed by individual interviews to explore nurses’ experiences and provide insight into the reasons for the diversity of the quantitative sample.

the qualitative findings help elaborate on or extend the quantitative results [18]. In this study, a mixed-method design to achieve the contextualization of information, taking a macro picture of moral distress and risk factors [15].

### A- quantitative stage

A descriptive study was conducted in this stage.

### Setting and participants in the quantitative stage

#### Setting

This study was conducted in surgical units (general surgery, neurosurgery, ophthalmology, ENT, orthopedics, and gynecology) and operating rooms of hospitals affiliated with Qom University of Medical Sciences.

The population included all nurses in surgical units and operating rooms (*n* = 224) who worked in teaching hospitals (*n* = 4) of the Qom University of Medical Sciences. The inclusion criterion was nurses in surgical technology, nurse anesthetists, and clinical nursing who worked at least a 6-month in surgical units and operating rooms. According to the formula

$$\left( {n = \frac{{{{({Z_{1 - \frac{\alpha }{2}}})}^2}{\sigma ^2}}}{{{d^2}}}{\text{Z}} = 1.96,\sigma = {\mkern 1mu} 0.9,{\text{ d}}{\mkern 1mu} = {\mkern 1mu} 0.15} \right),$$

the sample size was calculated at 160 nurses, and a 10% increase was added to the sample size. In this stage, 180 nurses in three subgroups of professions groups (surgical technology, anesthetist, and clinical nursing) were entered by stratified random sampling. Stratified random sampling was used when a population was divided into smaller subgroups based on members’ shared attributes or characteristics [19, 20].

#### Quantitative data collection

The questionnaire was distributed among all nurses who worked in the surgical units (general surgery, neurosurgery, ophthalmology, ENT, orthopedics, and gynecology) and operating rooms in the investigated hospitals. The researcher (A.H.) explained the study’s aims and obtained written informed consent from the participants. The nurses were asked to complete questionnaires by self-report. The researcher distributed 200 questionnaires among nurses in the professional groups and 180 questionnaires were completed. (Responding rate = 90%). The data collection period was from 20 May to 25 September 2023.

#### Study measures

In this stage, two questionnaires were used including demographic and Moral Distress Scale-Revised [21]. A demographic questionnaire was used to elicit information about the demographic and work-related characteristics of participants such as age, sex, clinical experience in years, marital status, academic degree (BSc, MSc), professions groups, and working shifts (fix, and rotation).

The data were collected by the Moral Distress Scale-Revised (MDS-R) questionnaire, which was revised by Hamric (2012) [21]. Ameri and colleagues (2015) validated this questionnaire in the Iranian context that was conducted in the present study (Cronbach’s alpha 0.92) [22]. The reliability of the questionnaire in the present study was calculated by internal consistency. (Cronbach’s Alpha = 0.87). The results of the factor analysis conducted by Soleimani and colleagues (2019) in the investigated context categorized the items into five categories including ‘role of healthcare providers’ (6 items), ‘futile care by healthcare team members’ (5 items), ‘working with unsafe colleagues’ (4 items), ‘condition of patients and their family’ (4 items) and, ‘limitations of healthcare system’ (2 items) [23]. Each of the 21 items is scored by participants in terms of how often the situation arises (frequency) and how disturbing the situation is when it arises (intensity). Thus, the scale for frequency ranges from 0 (never) to 4 (very frequently), and for intensity from 0 (none) to 4 (great extent) [21].

Regarding a relationship between repeated experiences of moral distress and the intensity of the phenomenon, the composite score for overall moral distress is computed in two ways. First, the frequency score and the intensity score are multiplied for each of the 21 items. This creates a new variable for each item, the frequency × intensity (f×i) score, which ranges from 0 to 16. Items rarely experienced or minimally distressing have low f×i scores, and items experienced frequently and as most distressing have higher f×i scores. Next, the composite score is obtained by summing each item’s f×i score. Overall moral distress scores for intensity, frequency, and level of moral distress were obtained from the total scores of questionnaire items (*n* = 21 items) in all three sections. Using this scoring scheme allows all items marked as never experienced or not distressing to be eliminated from the composite score, giving a more accurate reflection of actual moral distress. The resulting score based on 21 items has a range of 0–336 [21].

### Quantitative data analyses

Data were analyzed using SPSS version 21 software. The Kolmogorov-Smirnov test was used to determine the normality distribution of the data. Data were summarized by descriptive data including mean, standard deviation, number, and percentage. T-tests and Pearson’s statistical tests were also used.

### B- qualitative stage

#### Participants in the qualitative stage

In this step, the participants were selected by purposeful sampling. The nurses who achieved the highest and lowest frequency and intensity scores were invited to participate in the stage (*n* = 20). Purposeful sampling allows select participants who have experienced the phenomenon [24]. Maximum variation sampling aims to select cases that have the most significant successes or failures related to a topic of interest. The topic of interest was expected to yield valuable information from such extreme successes or failures [25]. The maximum variation sampling assisted in explaining different aspects of the phenomenon by the range of participants who experienced it and identifying similarities and differences of the phenomenon of interest [18]. This sampling helps to better understand the phenomenon. In this study, the method of maximum variation sampling was used to explore the experiences of nurses. The time and place of interviews were organized by agreement of the nurses. The aim and process of the study were explained and written informed consent in this stage.

#### Qualitative data collection

Semi-structured interviews were used to explore the nurses’ experiences. An interview guide was developed to direct the interviews. Three pilot interviews were conducted to test the interview guide in the initial step. Some questions were removed (e.g. could you please explain your perception of moral distress in the surgical units and operating rooms) and questions were reordered according to the results pilot. Interviews started with open questions and continued with probing questions. (Appendix 1) [26]. The interviews began with the main questions including ‘Have you ever experienced moral distress? how did you cope? what factors expanded the moral distress among nurses in the surgical units and operating rooms?’. A trained interviewer performed the interviews. Each interview lasted about 45 min. All interviews were recorded during the data collection. The data collection process continued until a rich interpretation was obtained, and no new code emerged during the interviews. (Saturation of results). The data were collected and analyzed in Persian and then translated into English for this paper. The results were translated and back-translated to English to ensure accuracy.

#### Qualitative data analyses

The conventional content analysis introduced by Graneheim and Lundman [27] was used to analyze data. The process of data analysis was done in 6 stages, including (1) transcribing of interviews, (2) extracting the semantic unit and open codes, (3) summarizing and classifying the open code and selecting an appropriate label for them as a sub-category, (4) sorting sub-categories based on comparing similarities and differences in categories, (5) selecting a suitable title with the ability to cover the resulting categories, (6) combining categories explaining the theme and choosing the appropriate label [27].

Based on this approach, meaning units were extracted from the statements of the participants that expressed their experiences. Then, the open codes emerged by taking notes in the margin of the text. The extracted codes were transferred to the coding sheet. Categories were extracted by organizing the codes based on the relationship between them. The themes were formed by comparing and contrasting the categories [27].

In this study, two qualitative researchers performed the data coding in a qualitative stage. The mean years of working experience in the field of qualitative research were 7 ± 3. The process was supervised by an expert who had expertise in qualitative research (working experience of 10 years). In cases of disagreement over the coding, discussions about the codes were continued until a consensus was achieved.

### Rigor

In this study, the criteria of credibility, transferability, dependability, and confirmatory were used to ensure trustworthiness [28]. The credibility criteria were achieved by using semi-structured interviews, field notes, and lengthy engagement with the research topic in the data collection, analysis, and interpretation steps. We have used some triangulation techniques in data analyses and interpretation. The confirmatory of the findings was ensured by reviewing the extracted codes and categories by all participants (member check), and the research team (peer check) in the steps of data collection, analysis, and interpretation. To do member-check, the interview texts and explored codes and findings were checked with all participants who contributed in the second stage to ensure that codes and categories were consistent with what they had experienced. Any adjustments were not made.

The external audit was conducted on the steps of data analysis, interpretation, and reporting. To do this, two experts (who were competent in qualitative research with a mean (SD) working experience of 12 [2] and age 54 [4] in SSU (Shahid Sadoughi University of Medical Sciences) checked the encoding process and forming of the findings (external audit). The current study explained a clear description of the context, and participant characteristics to facilitate the transferability of the findings.

Ethical Considerations: The present study was approved by the Ethics Committee of the National Agency for Strategic Research in Medical Education. Tehran. Iran. (ID: IR.NASRME.REC.1401.019). At the beginning of the interviews, the purpose of the research, the interview method, and the individuals’ right to participate or refuse to it in the study were explained. The participants were assured confidentiality of the recorded interviews and the collected information. Written informed consent was obtained from them.

## Results

Table 1 summarizes the demographic characteristics of the participants in the quantitative stage. According to the Kolmogorov-Smirnov Test, the distribution of the data was normal (P-value = 0.2). The mean of total moral distress scores ranged from 12 to 221, with a mean (SD) of 116.8 (42.73).

**Fig. 1:**
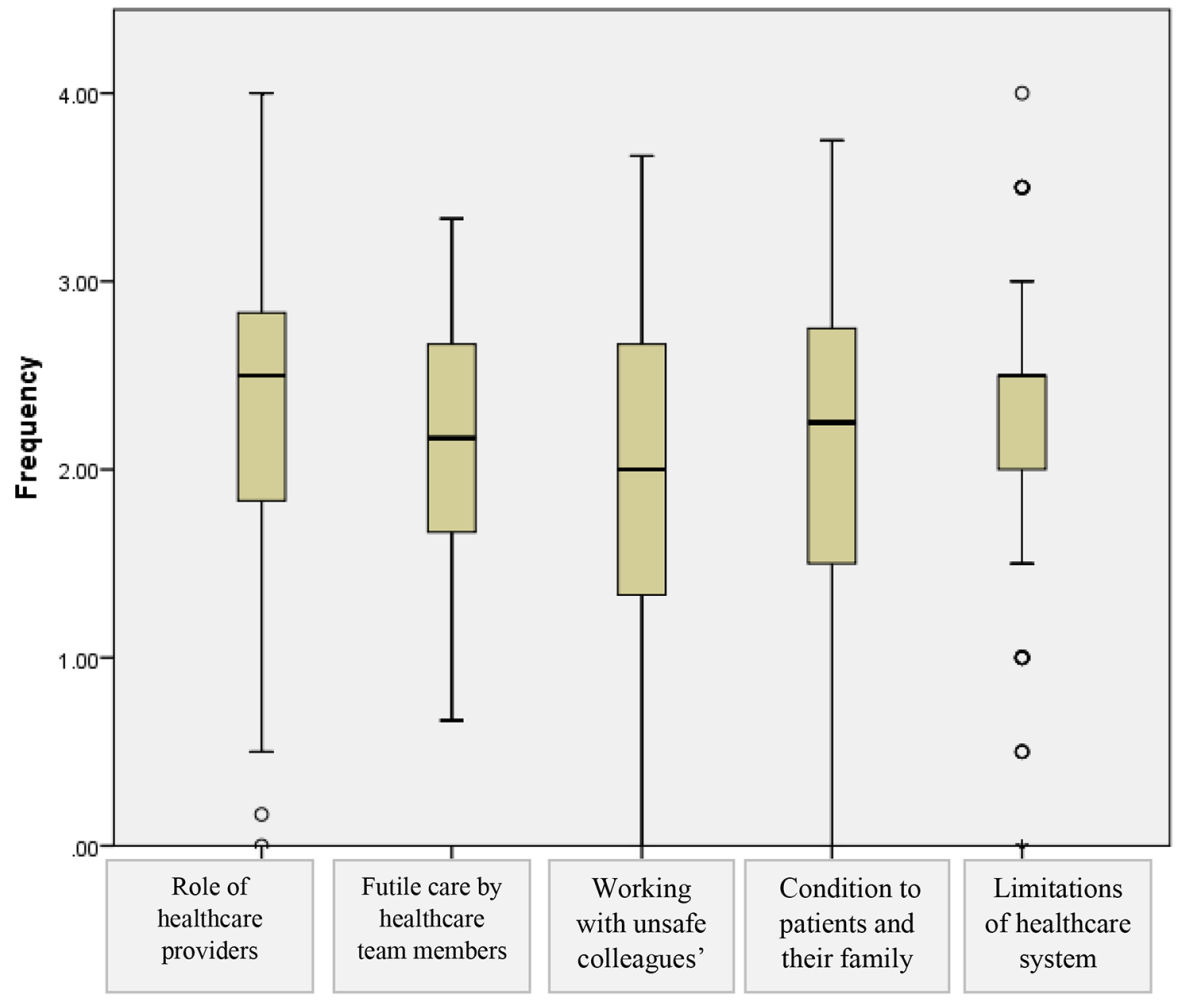
Main causes of moral distress on the Moral Distress Scale-Revised by mean item score for frequency (n =180). Note: Data spread is indicated by box and whisker plots, illustrating median, interquartile range and range of scores, excluding outliers

The causes of moral distress cited with the highest frequency related to the ‘role of healthcare providers’. (mean (SD) = 2.27 (0.79), range: 0.00–3.50). (Fig. 1).

**Fig. 2:**
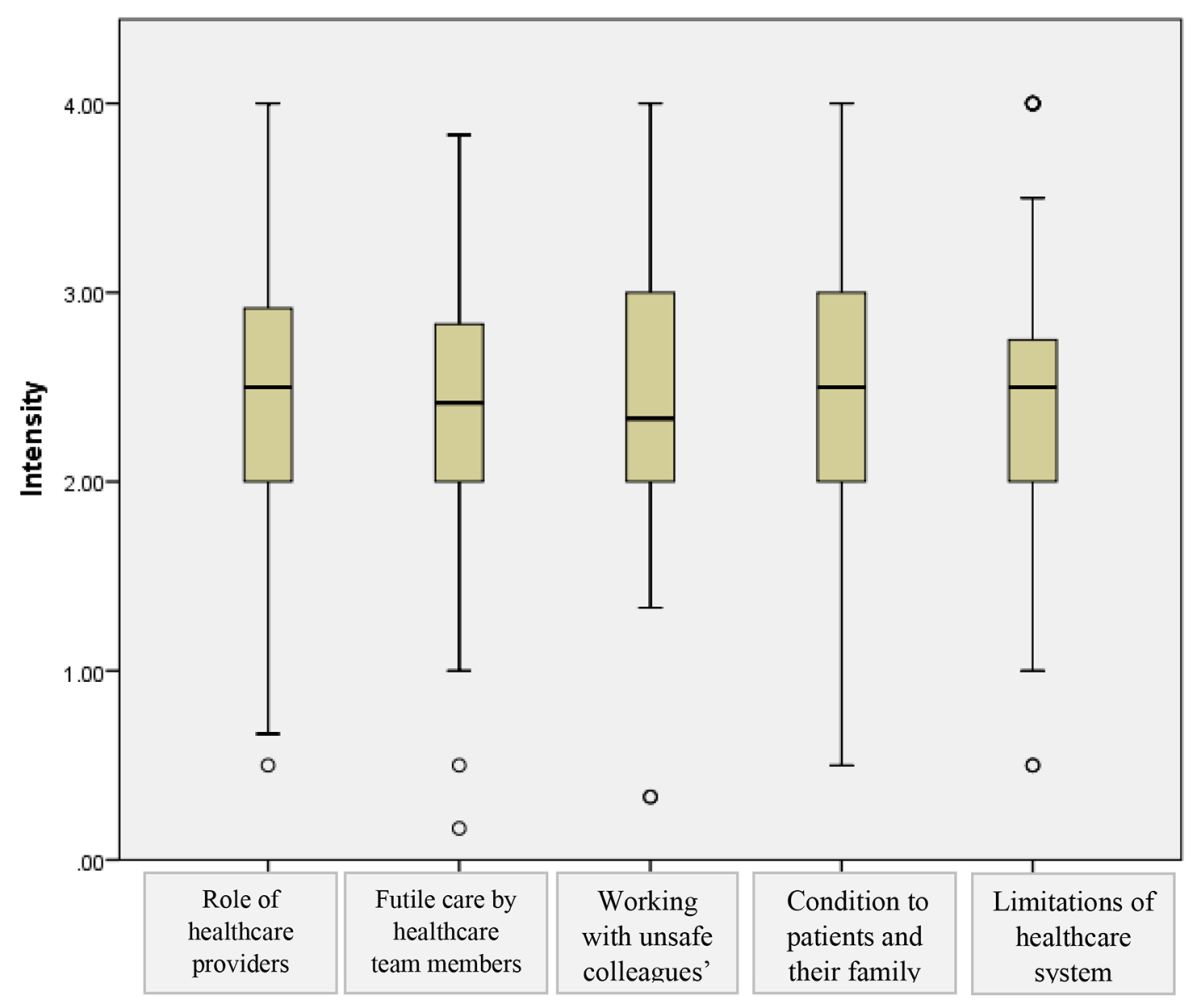
Main causes of moral distress on the Moral Distress Scale-Revised by mean item score for frequency (*n* = 180). Note: Data spread is indicated by box and whisker plots, illustrating median, interquartile range and range of scores, excluding outliers

Nurses reported high-intensity ratings for three domains relating to the ‘role of healthcare providers’ (Mean (SD) = 2.52 (0.75), range: 0.50–4.00), and ‘condition of patients and their family’ (Mean (SD) = (2.51 (0.69), range = 0.50 -4.00), ‘working with unsafe colleagues’ (Mean (SD) = 2.50 (0.71), range = 0.33- 4.00). (Fig. 2).

The results revealed mean (SD) intensity and frequency of moral distress were reported at 2.46 (0.54), range = 0.50–3.32 and 2.17 (0.58), range = 0.75–3.32, respectively. The results indicated that there is a significant relationship between the frequency and intensity of perceived distress of nurses. (*r* = 0.28, P-value = 0.01).

The most common sources of moral distress from the viewpoints of the nurses were reported in Table 2.

**Table 1:** Demographic Characteristics of Participants

		N (%) [*n* = 180]
**Gender**	Female	90 (50)
	Male	90 (50)
**Academic degree**	BSc	100 (55.55)
	MSc	80 (44.44)
**Marital Status**	married	93 (51.66)
	Single	86 (48.33).
**Working Shifts**	Rotation	98 (54.54)
	Fix	82 (45.45)
**Professions**	Surgical Technology Nursing	45 (25)
	Anesthetist Nursing	52 (28.88)
	Clinical Nursing	83 (46.11)
	*Mean (*± *SD)*	
**Age**	31.3 ± 5.9.	
**Working experiences**	5.56 ± 8.06	

There is a significant difference between the total score of perceived moral distress and nurses’ marital status (P-value = 0.04). The total scores among married nurses were significantly higher than among single nurses. There is no significant difference between the total score of perceived moral distress and nurses’ gender (P-value = 0.44), professions groups (surgical technology, anesthetist, and clinical nursing) (P-value = 0.22), groups of fix shift and rotation shift (P-value = 0.52) and their academic degree (P-value = 0.37). In addition, there is no significant relationship between the total scores of perceived moral distress of nurses with their age (*r* = 0.21, P-value = 0.05), and their working experience (*r* = 0.19, P-value = 0.08).

The results indicated that there is a significant relationship between the frequency of the perceived distress of nurses with their age (*r* = 0.30, P-value = 0.008), and their working experience (*r* = 0.32, P-value = 0.002). The frequency of nurses’ perceived distress among married nurses was significantly higher than among single nurses. (P-value = 0.01). There is no significant difference between the frequency of perceived moral distress and academic degree groups (P-value = 0.2), professions groups (surgical technology, anesthetist, and clinical nursing) (P-value = 0.1), and groups of fixed shift and rotation shift. (P-value = 0.15).

A significant difference between perceived distress intensity and gender was reported. The results indicated that female nurses reported more intensity of distress. (P-value = 0.04). The results indicated that there is no significant relationship between the intensity of perceived distress of nurses and their age (*r* = 0.04, P-value = 0.63), and working experience (*r* = 0.03, P-value = 0.76). There is no significant difference between the intensity of perceived moral distress and academic degree groups (P-value = 0.6), professions groups (surgical technology, anesthetist, and clinical nursing) (P-value = 0.33), and groups of fixed shift and rotation shift. (P-value = 0.7).

### Qualitative results

In this stage, 20 nurses participated. The profile of the participants is shown in Table 3.

**Table 2:** Most common sources of moral distress identified by nurses [n=180]

	Items	Mean	SD
**Intensity**	To work with nurses and other members of the team of healthcare providers who do not have the adequate competence to satisfy the patient’s care needs	2.63	1.08
	To work with other levels of nursing or care providers that, in my opinion, are unsafe	2.62	1.20
	To carry out the physician’s orders even in the cases when I know the test and required treatments are unnecessary (or the required tests and treatments…)	2.65	1.01
**Frequency**	I witnessed healthcare providers giving “false hope” to a patient or family	2.38	1.06
	Follow the family’s wishes to continue life support even though I believe it is not in the best interest of the patient	2.54	1.03
	I have witnessed that the quality of care is reduced due to poor team communication	2.45	1.22

**Fig. 3:**
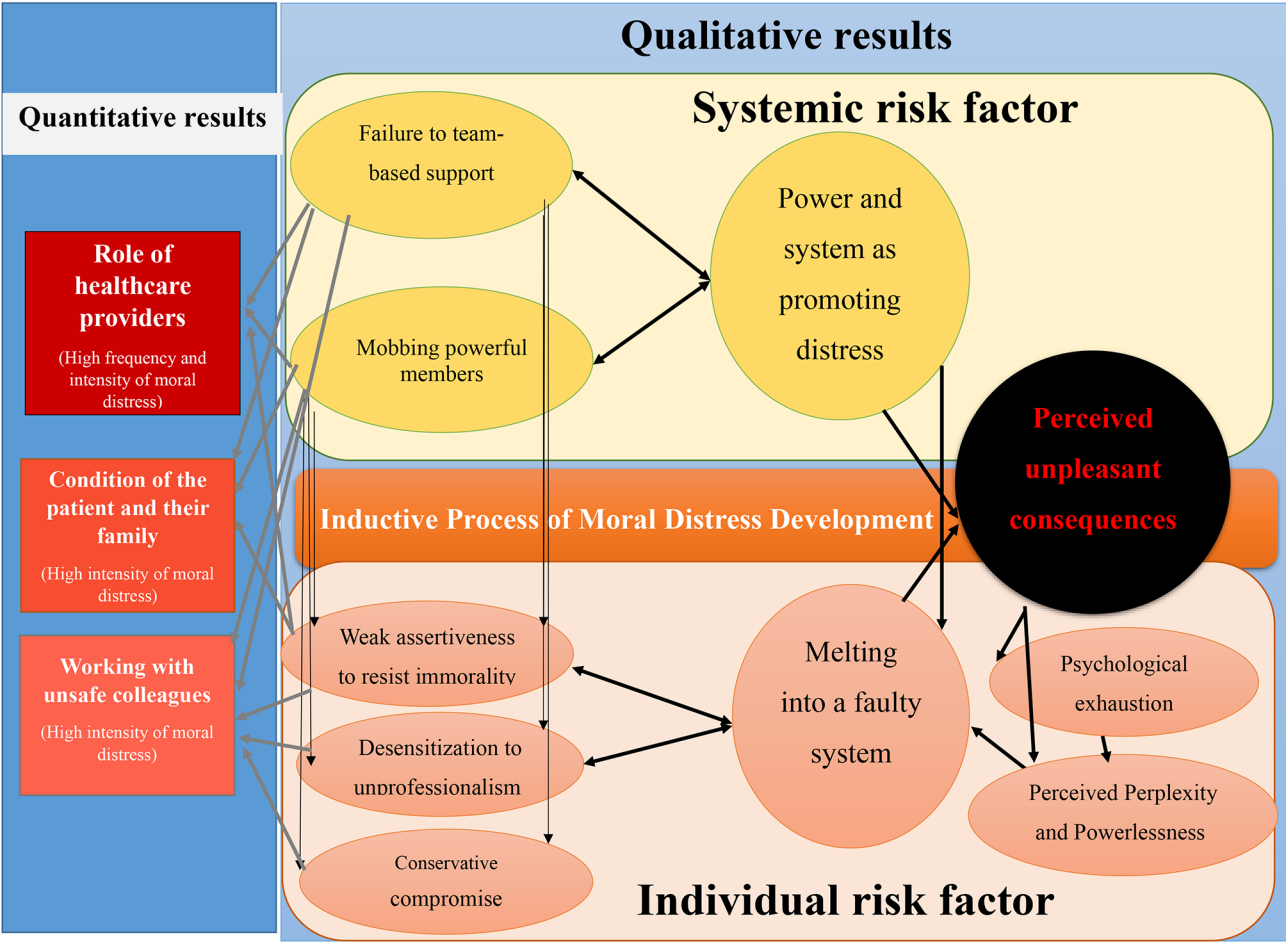
Mapping of Quantitative and Qualitative Results within Moral Distress Framework

The experiences of the participants were explored in the theme “inductive process of moral distress development” and were categorized into three categories: “melting into a faulty system”, “power and system as promoting distress” and “perceived unpleasant consequences”. (Fig. 3; Table 4).

**Table 3:** Profile of participants in the qualitative step

Profile	Gender	Age	Academic level	Total scores of Moral distress
				F×i
Participant No.1	Female	32	MSc	12.00
Participant No.2	Female	34	MSc	19.00
Participant No.3	Female	42	BSc	20.00
Participant No.4	Male	39	BSc	33.00
Participant No.5	Male	39	BSc	42.00
Participant No.6	Male	37	MSc	56.00
Participant No.7	Female	33	MSc	57.00
Participant No.8	Male	36	BSc	58.00
Participant No.9	Female	45	BSc	61.00
Participant No.10	Female	29	MSc	62.00
Participant No.11	Male	38	BSc	164.00
Participant No.12	Female	37	BSc	167.00
Participant No.13	Male	38	MSc	167.00
Participant No.14	Female	24	BSc	169.00
Participant No.15	Male	26	BSc	172.00
Participant No.16	Male	35	MSc	175.00
Participant No.17	Female	29	BSc	175.00
Participant No.18	Female	42	BSc	178.00
Participant No. 19	Male	33	MSc	191.00
Participant No. 20	Male	26	BSc	203.00

**Table 4:** Nurses’ experiences regarding moral distress in surgical field

Subcategory	Category	Theme
Conservative compromise	Melting into a faulty system	**Inductive Process of Distress Development**
Weak assertiveness to resist immorality		
Desensitization to unprofessionalism		
Mobbing powerful members	Power and system as promoting distress	
Failure to team-based support		
Psychological exhaustion	Perceived unpleasant consequences	
Perceived Perplexity and Powerlessness		

### A- melting in a faulty system

In this study, melting in the faulty system means the gradual process of desensitization of the nurses in the faulty system. It can be described as the way that nurses gradually accepted the unprofessional norms of the faulty system and did not react to unprofessional behaviors. After a while, this results in unprofessional behaviors to get along with other team members. The nurses’ experiences were categorized into three subcategories: conservative compromise, weak assertiveness to resist immorality, and normalization of unprofessionalism.

### A.1-Conservative compromise

Acceptance and silence were the key strategies used by nurses in dealing with moral distress. The participants mainly managed their distress by ignoring non-compliance with professional principles, accepting ethical challenges as a systemic norm, and not reacting to them to survive in the system. They considered silence and obedience to the unwritten rules of the system as the basic strategies for managing their distress. A nurse said:

*“If I had resisted in the system, the system would delete me. So, even if professional ethics were not respected, I would have to adhere to the norms of the defective system.” (Female, 32 years old).*

*“Our environment was full of contradictions that created moral distress. I just have to be silent and obey.” (Female, 34 years old).*

### A.2-Weak assertiveness to resist immorality

The participants stated that they did not dare to resist the unethical behavior. They were forced to remain silent and passive to protect themselves from the adverse consequences of protest and resistance. A nurse said:

*“Workers got in trouble between what they knew was right and what they should do! In the end, they had to do something against ethical and professional principles to avoid a new interpersonal challenge with the manager and other colleagues.” (Male, 39 years old).*

*“I noticed the doctor’s or resident’s mistake, but I could not even report it in the patient’s documents. Because the doctor may reprimand me. Sometimes, when the patient asks me after surgery, I have to lie to avoid the blame of doctors.” (Female, 42 years old).*

### A.3-Desensitization to unprofessionalism

Turning unethical behaviors into the norm in the system led to the workers desensitized and preferring to be passive in distressing situations. A nurse stated:

*“I experienced so much moral misconduct, errors, and contradictions during my career. Thus, I am accustomed and it becomes normal for me.” (Female, 33 years old).*

*“The unprofessionalism norms affect our behavior, even the behavior of students who were studying at this hospital.” (Male, 39 years old).*

*“I used to be unprofessionalism that very annoyed with me at the beginning of working, but now it seems to be normal. It seems that if I do the right thing, I will be the one who disobeys that routine or unwritten rule.” (Female, 45 years old).*

*“In the surgical department, the employees faced a lot of moral distress, but after working for a few years, many things became normal for them. At the beginning of work, they adhere to professional principles and ethics, and if they make a mistake, they will be upset. But when they did not get any support, they did not feel bad about the unprofessional behaviors that they did.” (Male, 37- years old).*

### B-Power and system as distress promotor

In this category were discussed the risk factors of distress at the system level. The nurses believed that the mobbing of the powerful members of the team, the dominance of the medical paternalistic approach in the healthcare teams, and the lack of support for resistance against the mobbing and immorality of others, caused the development of distress among the nurses.

### B.1-Mobbing powerful members

In this subcategory, the nurses experienced mobbing from powerful people. They become powerful because of their role in the team (e.g., leader and supervisor), professions (e.g., surgery), and work experience (e.g., senior). The nurses believed that the powerful people forced other team members to do activities that were not aligned with the guidelines or ethical and professional principles. These were experienced as risk factors of distress among nurses. A nurse stated:

*“Workers who become powerful in the system because of their background or job position, ask me to do things that were not in line with moral principles. I have to do that because of fear that they blame me…” (Male, 36 years old).*

*“The main task of a surgical technologist was to count gases before, during, and after surgery. Many surgeons in these hospitals said that it was not necessary, and this became their routine manner. If I did not follow that routine, when an error was reported, I was blamed and sometimes even ridiculed. In a case, where gases remained in the patient’s abdomen, the surgeon blamed me. Finally, I was recognized as guilty.” (Female, 29 years old).*

*“I suffered from hidden powers that did not allow me to do the task professionally. These factors caused distress for me.” (Female, 37 years old).*

### B.2-Failure to team-based support

In this subcategory, the lack of support was explored as a risk factor in distressing situations. The nurses stated that when unethical challenges emerged no one supported them neither team members in the interprofessional or uni-professional members nor the supervisors and managers. No one resists mobbing and non-compliance with professional principles.

*“When I want to resist this unprofessional request of the surgeon, instead of my supervisors and colleagues supporting me, they keep silent and sometimes blame me.” (Female, 26 years old).*

*“When there was a challenge, the lack of teamwork among the workers, and the lack of support from the supervisors led to workers experiencing a bad psychological burden.” (Male, 26 years old).*

*“I did not report professional and ethical errors and challenges to my supervisors. Sometimes, I was blamed by my supervisor due to my error reporting.” (Male, 38 years old).*

### C-Perceived unpleasant consequences

In this category, the perceived unpleasant consequences of the nurses were explained at the individual and systemic levels. The nurses’ experiences were categorized into two subcategories including risks to well-being and mental-psychological problems, and perceived perplexity and powerlessness.

### C.1-Psychological exhaustion

Emotional exhaustion was explored as a consequence of moral distress.

*“A new mother died under my care due to a resident’s medical error. A mother who had just given birth to a baby and she had two children at home. I was upset that day, and now after several months, I still haven’t calmed down. Whenever I remember it, I get upset.” (Female, 29 years old).*

*“In many of these cases, the experienced distress unfortunately remains unresolved. These made a twinge of conscience that stays with me everywhere in the workplace, at home, and for the rest of my life.” (Female, 42 years old).*

*“I can’t bear it when I see the dishonesty of workers, and I suffer from psychological problems like anxiety.” (Male, 35 years old).*

The participants believed that physical and mental exhaustion resulted in distress among them and distress led to their mental problems.

*“Excessive fatigue and unpleasant physical and mental status led to my procrastination. This procrastination led to mental exhaustion for me.” (Male, 34 years old).*

*“I know professional principles, but I did not work the best for patients. Because I was tired at that moment and I’m not in a good physical and mental status”. (Female, 24 years old).*

### C.2-Perceived perplexity and powerlessness

The participants believed that frequent exposure to moral distress has created a sense of futility and disillusionment among them. They started when we knew what the right thing was, but we could not do the right thing, we were confused. They chose the behavior that caused the least damage to them and was more compatible with the system norm.

*“In this case, I did not allow unprofessional behavior to be conducted in the operating room. After a few days, I realized that the surgeon complained to my supervisor. I received a reprimand. I felt upset. A sense of emptiness and confusion! Why was I reprimanded, and what is right? What was my mistake?” (Male, 33 years old).*

*“Once I saw an unethical behavior in my colleague and warned him in a friendly way. Then, I realized that he reported this. Finally, I have been exiled to another unit. I felt disillusioned for many times”. (Male, 37- years old).*

## Discussion

The results indicate a moderate to moderately high level of moral distress which is what the MDS-R measured. The causes of moral distress cited with the highest frequency and intensity related to the ‘role of healthcare providers’. The experiences of the nurses in the theme “inductive process of moral distress development” were categorized into three categories: “melting into a faulty system”, “power and system as a promoter of distress” and “perceived unpleasant consequences”.

The present results of high frequency and intensity of moral distress sources were reported in the category of ‘role of healthcare providers’. The inductive process of moral distress development through team members and system elements was explored in the qualitative step. In line with our findings, Burton in a mixed-method study synthesized quantitative and qualitative findings. Their results showed the nurses have been exposed to intensely stressful and morally injurious events. Their quantitative findings showed organizational support and institutional betrayal predicted moral distress among nurses. They discussed the main sources of nurses’ moral distress including external constraints (organizational support, traumatic strain, institutional betrayal), clinical situations (inadequate resources, workplace social support, peer support, conflicts with administration), and internal constraints (feeling powerless, repeated exposure to trauma, patient suffering). The nurses indicated feeling disregarded by management and institutional structures increased the nurses’ intention for a plan to leave bedside practice [16]. Likewise, Prompahakul and colleagues in a mixed-method study showed powerlessness explored as the main source of moral distress of nurses in the Thailand context. They indicated sources of powerlessness were evident at the patient/family level, the team level, and the organizational level. They showed working with incompetent colleagues, poor communication and collaboration, excessive documentation requirements, and lack of resources were explored as morally distressing at the team and system levels [17]. Boulton and colleagues in a mixed-method study indicated moral distress was widespread among UK ICU professionals and resulted in a negative effect on patient care, professional wellbeing, and staff retention. They indicated experiencing moral distress led to a range of negative emotions and behaviors such as frustration, upset, deflation, avoidance of interaction, and dissatisfaction among nurses [26]. Similarly, perceived unpleasant consequences of the nurses in the present study were categorized into sub-categories of psychological exhaustion, and perceived perplexity and powerlessness.

The high frequency and intensity of moral distress of nurses who work in operating rooms and surgical units were purported in the domain of the ‘role of healthcare providers’. The domain assessed the causes of moral distress when diminished patient care quality due to poor team communication, lack of provider continuity, and work with incompetent providers. In this domain, the items of ‘working with incompetent team members’ and ‘working with unsafe providers’ resulted in the highest intensity of moral distress. Also, the item of ‘poor team communication’ led to highly frequent distress in the operating rooms and surgical units. The present study conducted in educational hospitals and the participation of novice students in surgical teams may affect the moral distress of nurses. According to the qualitative findings, the failure to team-based support at the interprofessional and the uni-professional levels was explained moral distress of nurses. The contextual challenges of team-based culture and the cooperation in the investigated hospitals may result in moral distress to nurses. Numerous studies also reported weaknesses in teamwork and interprofessional cooperation in the investigated context [29–31], which is similar to the results of the present study. According to the current explored model, the inadequacy of team-based support systems led nurses to use a compromise strategy to cope with unprofessional behaviors and distressing situations. Progressively, they desensitized and melted into the faulty systems and enhanced the adverse consequences of moral distress such as execution and powerlessness perception. These consequences eliminated nurses’ assertiveness and created unprofessionalism norms. In line with our results, Millis identified the moral distress in the surgical team was influenced by the level of support from co-workers and working alongside inadequately trained colleagues [14]. Poor communication among team members was identified as a promoting factor for moral distress [5, 32]. Similarly, Lusignani’s study indicated that helping unqualified doctors was the highest cause of distress among nurses in medical, surgical, and intensive care units [13]. Likewise, Woods’ study indicated that the main perceived distress of nurses in New Zealand included the inappropriate treatment of unqualified colleagues. They believed the lack of support from the system was the most important cause of perceived challenges [33].

The high intensity of items in the domain of ‘working with unsafe colleagues’ was reported. This domain addressed distress such as assisting a physician who providing incompetent care, avoiding taking action in a medical error situation and an ethical issue, and carrying out unnecessary treatments. In line with our results, Francis’ meta-analysis study indicated that the culture of continuing invulnerability and non-disclosure of errors was an unknown and important distress for many healthcare workers. The lack of systemic support and the weakness of team mechanisms for support against distress were explained in Francis’s study [34]. In the present study, the failure of team-based support, mobbing of powerful team members, as a systemic factor, and conservative compromise of workers and desensitization to unprofessionalism as an individual factor were explained in the quantitative findings. The nurses explained the dominance of medical paternalistic norms, and the hierarchical approach among the members caused the members with less experience or allied medical professions to become second-class citizens in the system. This norm directed nurses to use an emotion-oriented coping response through compromise. This norm resulted in stabilizing the unprofessional behaviors of seniors and increasing the induced passiveness of nurses. These affected the melting of nurses in the faulty system and the elimination of resistance in distressing situations. In addition, the lack of support from the interprofessional team members, supervisors, and system managers made nurses learn how to cope with challenging situations through silence and passiveness. According to the explored model, this process resulted in an increase in the number of distressing situations and unpleasant consequences at the individual level. The perceived psychological exhaustion and the perceived powerlessness had a negative impact on the individual factors of distress and facilitated a reduction of assertiveness and passiveness among nurses. In line with our results, McCarthy and colleagues declared identifying nurses as a group of health professionals whose voices were ignored or marginalized resulted in disempowered nurses and encouraged them to avoid their moral responsibilities [1]. Francis indicated that the lack of support mechanisms during difficult situations made providers feel isolated, which often led to pessimism and the adoption of negative coping strategies such as detachment to manage their distress. The adverse effects of job distress increase the potential of job burnout, feelings of isolation, and feelings of incompetence [34]. Mobbing as an antecedent factor was explored in this study that creates distress and adverse results such as reducing the psychological well-being of nurses. Behaviors such as doing nothing, moving away, focusing on work and working harder, being silent, and tolerating and normalizing mobbing behavior were common responses [35].

The domain of ‘condition of the patient and their family’ achieved a high rank of moral distress intensity in the viewpoints of nurses. A disagreement between nurses, physicians, and patients’ families regarding patient management was a key factor that may result in moral distress. The domain addressed items such as following the family’s wishes for the patient’s care because of fears of a lawsuit, and providing care because of the physicians’ fears. The qualitative results indicated that the failure of team-based support had an important role in the creation of distressing situations. Team atmosphere, organizational culture, implicit and explicit powers in the system, and the weakness of nurses’ assertiveness affect the frequency of perceived distress in difficult situations. The weak support from members in interprofessional and uni-professional teams in difficult situations caused distress among nurses, which was explained in the category of failure team-based support. In the present context, the lack of a participatory decision-making process and the opportunity to express nurses’ opinions caused them to eliminate their assertiveness to express opinions [29, 30, 36]. These challenges enhanced the perception of powerlessness and directed to passives among nurses. The previous studies revealed the physician paternalistic approach in the present context resulted in the elimination of the participation of other team members such as nurses in the decision-making process [29, 30, 36]. Similarly, Giannetta in a systematic review study indicated that the weak support of supervisors was one of the effective factors in the perceived distress of nurses [37].

The present results indicated that the intensity of the perceived distress of female nurses was reported as higher than men. However, there is no difference in terms of frequency and total scores. In line with the present study, O’Connell et al. indicated that female nurses experienced higher distress [8]. In Giannetta’s review study, gender was determined as an effective factor in perceived distress. The female nurses who worked in incentive care at hospitals experienced more distress [37]. The perceived psychological burden and vulnerability of women imposed on them can justify these results. In addition, married nurses experienced moral distress more in comparison to single ones. The result of Ness and colleagues [38] was in line with our findings. In our study, married nurses were more vulnerable due to multiple duties at work, personal life, and life stress. The limitation of support in individual life and support of colleagues and team managers may result from married nurses experiencing more distress. Asadi (2022) indicated that marital status was significantly associated with moral distress. They revealed married nurses experienced less moral distress. The results differed from our findings [39]. Cultural differences and family support systems may influence the findings.

The conceptual model of risk factors of moral distress was explained based on the experiences of the nurses in the surgical units and operating rooms. In this model, the risk factors of moral distress were explained in two sections, individual factors and systemic factors. Systemic factors are classed into ‘failure to team-based support’ and ‘mobbing powerful members’. These subcategories were categorized in the ‘power and system as promoting distress’ category. Based on the experiences of the nurses, this category had an impact on individual factors and the creation of unpleasant consequences of distress. In the individual section, three subclasses were explained, which include ‘conservative compromise, weak assertiveness to resist immorality and desensitization to unprofessionalism’. These subclasses created the ‘melting into a faulty system’ category, which directly affected ‘perceived unpleasant consequences’. Two subcategories of unpleasant outcomes including ‘psychological exhaustion’ and ‘perceived perplexity and powerlessness’ were experienced as a result of moral distress by nurses. The subcategories of ‘perceived unpleasant consequences’ had a negative effect on individual factors. The nurses’ perception of powerlessness and perplexity was directed to their passiveness in the team activities. These factors accelerate the melting of nurses in the faulty system by reducing assertiveness and directing them to use the conservative compromise strategy. Therefore, the unpleasant consequences resulting from distress situations were identified as both the consequence and the accelerators of distress in surgical units and operating rooms from the viewpoints of nurses. Based on the present results, individual factors as the most vulnerable factor in causing distress were affected by the systemic factors and consequence factors of distress.

### Implication of findings

The qualitative results revealed systemic and individual factors explicitly or implicitly led to the nurses preferring the use of emotion-focused coping responses such as silence and compromise. Thus, it is recommended to create support mechanisms related to the psychological health of nurses and to develop their capabilities in interpersonal and interprofessional interactions, assertiveness, and the use of suitable coping strategies in distress situations. It is also suggested to create team-based support, such as the development of team-based care mechanisms, and interprofessional team meetings to debate medical error, unethical behaviors, and moral distress in the operating rooms and surgical units. Moreover, mechanisms of feedback and medical error disclosure assisted the nurses in moral distress situations [14]. The construction of a culture of psychological safety and teamwork was recognized as the key mechanism for protecting against burnout and adverse outcomes of moral distress. In addition, the opportunity for debriefing, mentoring, and reflective practice as valuable methods suggested to enable nurses to reduce the impact of psychological injuries of moral distress [34]. Boulton and colleagues suggested supportive environments and supportive interventions according to the individualistic nature of coping with moral distress [26].

Limitation: the sample size and use of self-report to collect data in the quantitative stage and limitations of the qualitative method in one university restricted the generalizability of the results. In the quantitative stage is at risk of selection bias. Those experiencing high levels of moral distress may have not tended to contribute and relive their experiences, or those with low levels of moral distress may not appreciate its value and not participate. This study is a snapshot and effect of how the nurses were feeling, or what clinical cases were faced on their unit. Moreover, this study was conducted in operating rooms and surgical units, which can be different from the perceived distress of nurses in other hospital units.

## Conclusion

The results indicated that the frequency and intensity of nurses’ moral distress in the operating room and surgical units ranged from moderate to moderately high levels, respectively. The “induction process of distress development” was explored as the experiences of the nurses related to risk factors of moral distress. This process was a cycle under the influence of systemic and individual factors. The failure of team-based support and the norm of mobbing powerful members of the system were explored as systemic risk factors of distress. Individual factors including weak assertiveness to deal with distressing situations, desensitization to unprofessionalism, and using a conservative compromise strategy were influential in experiencing distress among nurses. The individual and systemic factors caused perceived unpleasant consequences, including perceived perplexity powerlessness, and psychological exhaustion. The perceived unpleasant consequences facilitated the prevalence of distress by melting nurses into the faulty system in the operating rooms and surgical units.

### Electronic supplementary material

Below is the link to the electronic supplementary material.

Supplementary Material 1

## Data Availability

The datasets generated and/or analyzed during the current study are not publicly available due to the confidentiality of the data of participants but are available from the corresponding author at reasonable request.
