# Supplementary material for: Inductive process of moral distress development in viewpoints from surgical nurses: a mixed-method study
Source: BMC Nurs. 2024 Mar 21;23:191. doi: 10.1186/s12912-024-01786-3 (PMC10956303; doi:10.1186/s12912-024-01786-3)
Supplement: Supplementary file 1 — Supplementary Material 1 [file 12912_2024_1786_MOESM1_ESM.docx]

| **Appendix 1- Interview guide** | |
| --- | --- |
| **Introduction:** | - Introduce self and project. - Please explain your role and professional background: Age, professional role, and years of work. |
| **Ethical consideration** | - Confirm confidentiality: Confirm that individual comments will not be discoursed with others (employers and seniors). - Ensure written informed consent. Advice of audio-recording could you just confirm that you are happy to be audio-recorded. - The research team is independent of the hospital management and working with Education Development Center and the data is protected. - We are interested in their experiences and views and would like them to be as honest as possible. - I may make the occasional note during the interview. |
| **Questions** | - What kind of things make you feel uncomfortable at work? - What kind of things do you find challenging or upsetting at work? - Have you had a situation where you felt that your personal beliefs were in conflict with what a patient wanted? With what the team wanted? When you experience these kinds of situations, how do you cope? Do you discuss them with anyone? - When you experience these kinds of situations, how do you cope? - Have you experience the support of doctors/nurses facing the distress situations? - What do you think about moral distress root causes? What do you think about the most troublesome sources of moral distress? - What factors expanded the moral distress among nurses in the surgical units? |
| **Probing questions** | - Can you give me a specific example? - How did it make you feel? - What did you do? - Can you describe how this made you feel? - What were your thoughts and feelings during and after this? - What was helpful? |
